# The Relevance of Heart Rate Variability for Hypnotherapy and Psychotherapy

**DOI:** 10.3390/brainsci16040352

**Published:** 2026-03-25

**Authors:** Donald Moss

**Affiliations:** College of Integrative Medicine and Health Sciences, Saybrook University, Pasadena, CA 91103, USA; dmoss@saybrook.edu

**Keywords:** heart rate variability, vagal activity, autonomic regulation, emotion regulation, psychotherapy, hypnotherapy

## Abstract

**Highlights:**

**What are the main findings?**

- Heart rate variability (HRV) is a robust biomarker of autonomic regulation, closely linked to vagal activity, prefrontal cortical function, emotional regulation, and resilience, and serves as a transdiagnostic marker of both medical and psychological disorders.
- Higher HRV is consistently associated with characteristics essential for effective psychotherapy and hypnotherapy—including social engagement, compassion, sense of safety, autonomic balance, and cognitive flexibility—while lower HRV predicts morbidity, mortality, and psychopathology.

**What are the implications of the main findings?**

- Integrating HRV-enhancing strategies—such as HRV biofeedback, resonance frequency breathing, slow-paced respiration, meditation, exercise, music-based interventions, nutritional modification, and self-hypnosis—can optimize readiness for and responsiveness to psychotherapy and hypnotherapy.
- Therapists can deliberately harness brain–heart regulatory mechanisms to strengthen treatment outcomes, positioning somatic regulation and vagal activation as foundational components of integrative psychotherapeutic practice.

**Abstract:**

This review examines what constitutes heart rate variability (HRV), the relationship between HRV and the autonomic nervous system, and the physiology driving HRV. HRV is correlated with vagal nerve activity and parasympathetic nervous activation. Higher HRV is correlated with youth, active lifestyle, adaptive capacity, and good health. Next, the review examines the medical significance of HRV, especially the correlation between lower HRV and the presence of medical and psychological disorders. In general, HRV serves as a biomarker for health and disease, an index of autonomic nervous system dysregulation, an index of prefrontal cortical functionality, and a marker for psychopathology across diagnoses. Higher HRV is associated with several characteristics associated with successful psychotherapy and hypnotherapy: social engagement, compassion, emotional regulation, and cognitive flexibility. Given this association, somatic regulation should be regarded as integral to treatment alongside psychotherapy and hypnosis. Understanding HRV can enable the psychotherapist and hypnotherapist to optimize treatment. In effect, the therapist can harness the power of the brain and nervous system to better prepare the patient for therapy and to enhance the process of therapy. This review encourages therapists to utilize several strategies and interventions to increase patients’ HRV levels prior to and during therapy. The review will be most applicable for those hypnotherapists who integrate hypnosis into counseling and psychotherapy. The review describes the process by which HRV biofeedback training guides the individual to voluntarily increase HRV. It also identifies a number of lifestyle parameters and self-care practices (including self-hypnosis) that increase HRV. Encouraging lifestyle and self-care practices to increase HRV can support a greater response to hypnotherapy and psychotherapy. With additional training, hypnotherapists can integrate HRV biofeedback into a hypnosis practice. Further, several simple interventions already within the scope of most hypnosis practitioners can be utilized to enhance HRV at the beginning of a hypnotherapy process, and again during the process of therapy.

## 1. What Is Heart Rate Variability?

Heart rate variability (HRV) refers to the moment-to-moment change in the time interval between adjacent heartbeats. The interbeat interval, the time between R-waves in milliseconds, is the basic unit from which all heart rate variability statistics are derived. The R-wave is the sharp upward space visible in the electrocardiogram. The interbeat interval is often called the R-R interval; the two terms are interchangeable. Research on heart rate variability begins with an array of interbeat intervals. Table 1 shows several time domain HRV statistics that are calculated from the interbeat intervals [1].

HRV can be measured by either an electrocardiogram (ECG), which measures the electrical activity of the heart or a photoplethysmograph (PPG), which measures the blood pulse volume in the cardiovascular system. The PPG estimates the time of the heartbeat from the moment of maximal amplitude in the pulse wave and may diverge from the ECG, especially during movement, vasoconstriction, arrhythmia, or any times of poor signal quality. HRV data derived from the ECG is more precise and is preferred in research. In either case, HRV is calculated from the interbeat intervals, the time periods between R-waves on the ECG or the peak pulse wave on the PPG. A healthy individual will display greater variability in those interbeat intervals.

## 2. Variability Equates to Health and Resilience

A healthy heart is not a metronome [2]. Healthy, resilient physiological systems show large oscillations in heart rate. Youthful, aerobically active individuals show higher variability in heart rate [3,4,5]. As the heart loses its variability, the risk for illness and death increases, whereas increased variability is associated with health, resilience, and optimal functioning. Decreased variability typically accompanies the aging process, sedentary lifestyle, and illness [6,7].

## 3. Neurophysiology of HRV

Heart rate variability serves as an index for neuro-cardiac function. The variability is generated by heart–brain interactions and dynamic autonomic nervous system (ANS) processes [1]. HRV is strongly correlated with parasympathetic activation. The variability of heart rate can be analyzed into frequency ranges, including ultra-low frequency (ULF), very low frequency (VLF), low frequency (LF), and high frequency (HF) variability [8] (See Table 2). In other words, a frequency analysis isolates heart rate changes that cycle within specific time ranges. Thus, HF-HRV involves heart rate changes cycling between 0.15 and 0.4 times per second.

## 4. Measurement Conditions for HRV Activity

The two slowest frequency bands, ULF and VLF, are so slow that they cannot be reliably assessed in brief samples of heart rate data. All four bands can be assessed by 24 h monitoring, as is used in cardiological diagnostics. The values found in short-term measurements are typically lower and cannot be compared reliably with 24 h values.

The power of HRV in the high frequency range (HF-HRV) reflects respiratory-linked vagal modulation of heart rate, when measured in a resting condition (Power is a measure of the signal energy within each frequency range. The various ways of measuring power are described by Shaffer and Ginsberg (2017) [1]). It is sensitive to breathing rate. Therefore, for research, HF-HRV should be measured at normal breathing rates, not at a slower training breath rate. Data recorded during HRV training periods will show increased activity and power in the low frequency range of HRV, an artifact of slow paced respiration.

HF-HRV, when measured in resting conditions and at normal breathing frequencies, is a marker for vagal nerve activation [2,9,10]. The vagus nerve is a long winding and wandering cranial nerve that regulates many organs throughout the body. It forms a two-way highway between gut and brain, as well as heart and brain, with many more connections. The vagus nerve provides the majority of the fibers for parasympathetic nervous system activity [11,12,13].

Heart rate variability (HRV) and vagal activation are key biomarkers linking emotional resilience, medical health, and physiological regulation. Increased parasympathetic activation (or vagal nerve input) increases the moment-to-moment oscillations in heart rate and increases both health and performance benefits. Accordingly, HRV is an emergent property of multiple interdependent regulatory systems in the body which operate on different timescales to “help us adapt to the environment and to psychological challenges” [1].

The systems that generate HRV span the neuro axis from the prefrontal cortex and the insular cortex to the intrinsic cardiac nervous system and the medulla oblongata, which function as integration centers [14].

## 5. Respiration, Autonomic Nervous Balance, and Heart Rate Variability

Respiration and autonomic balance contribute to heart rate oscillations. Respiration is a core component driving changes in heart rate. During each in-breath, heart rate increases. After a delay of seconds, blood pressure rises. The baroreceptors, the receptors that sense the increase in blood pressure, fire more rapidly during the out-breath and heart rate decreases (The baroreflex or baroreceptor reflex is a fast-acting feedback mechanism that maintains stable blood pressure by detecting changes in arterial wall tension. The baroreflex produces much of the heart rate fluctuations in the low frequency range.). Blood pressure falls a few seconds later. These dynamics are explained in greater detail in two publications by Lehrer and Gevirtz (2014) [15] and Lehrer and Vaschillo (2008) [16]. This synchrony of respiration and cardiac activity is called the respiratory sinus arrhythmia (or RSA) [17]. Respiratory sinus arrhythmia may sound to the reader like pathology; however, it is a normal regulatory process within healthy physiology.

## 6. What Is the Respiratory Sinus Arrhythmia?

**Figure 1:**
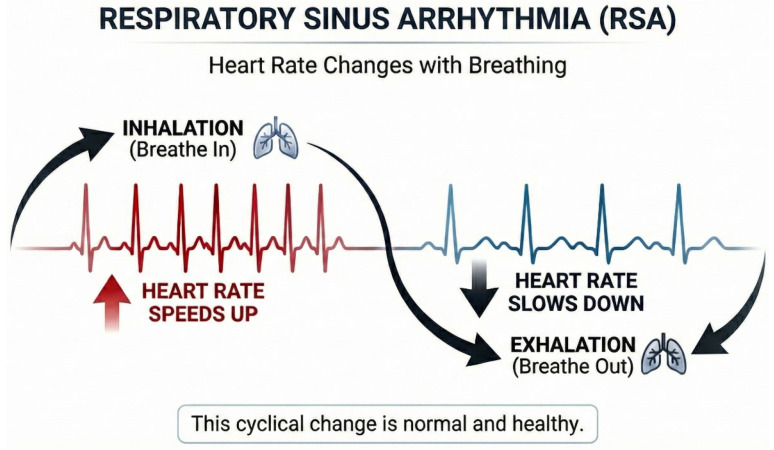
Respiration Sinus Arrhythmia: Heart rate speeds during inhalation and slows during exhalation. Graphic used courtesy of BioSource Software (Kirksville, MI, USA), HRV Biofeedback Tutor.

During inhalation, vagal nerve activity withdraws, producing increasing heart rate, allowing the intrinsic cardiac pacers to drive the heart rate increase. This is a parasympathetically mediated process. In turn, the vagal nervous system re-engages during exhalation and acts as a brake, slowing heart rate [18] (See Figure 1).

## 7. Heart Rate Variability: The Resonance Frequency

Breathing at a specific rate called the resonance frequency produces the largest, organized oscillations in heart rate [19,20]. The resonance frequency varies by individual but approximates to six breaths per minute. The individual’s baroreceptor loop is the homeostatic mechanism governing blood pressure; it resonates close to this same six-cycle-per-minute frequency. Accordingly, the resonance frequency is the person-specific breathing rate at which the baroreflex and the breathing rate correspond. In effect, breathing at that rate employs two biological forces—respiration and the baroreflex—and together, they produce larger oscillations in heart rate. This effect cannot be produced simply by “slow breathing” or “deep breathing,” without attention to the breathing rate. Training individuals to breathe at this resonance frequency, which produces the largest oscillations in heart rate, has produced moderation of symptoms in a variety of medical and psychological disorders [21,22]. We will discuss the resonance frequency further, as well as the process for its measurement, in the discussion of HRV biofeedback training.

## 8. The Importance of Heart Rate Variability in Medical and Psychological Health

Heart rate variability, what difference does it make? Since the 1970s, biomedical research evidence has accumulated, showing that lower heart rate variability accompanies and even precedes many illnesses and health problems. It is a biomarker, predictive of disease states and disease progression. Research shows a strong relationship between lower HRV and disease states in chronic medical illness—such as diabetes, heart disease, hypertension, and cancer [23,24,25,26,27].

HRV is similarly predictive of mental health problems, especially anxiety, depression, and post-traumatic stress disorder [28,29,30,31,32,33] summarized the extensive evidence for lower HRV in most psychological disturbances. Research consistently shows the presence of low HRV in several groups with psychological disturbance, including anxiety disorders, depression, post-traumatic stress disorder (PTSD), borderline personality, bipolar disorder, and schizophrenia. The link between low HRV and emotional/mental disturbance is so great that Beauchaine and Thayer (2015, p. 345) [34] proposed that HRV is a “transdiagnostic marker of psychopathology.” In other words, low HRV predicts a heightened risk of emotional problems across psychiatric diagnostic categories [35]. In contrast, higher HRV appears to present a buffer against psychological disturbance [33].

Research studies have also found evidence that HRV is reduced in several other psychological disorders, including borderline personality disorder [36], bipolar disorder [37], schizophrenia [38], and substance use disorder [39].

In summary, individuals with many psychological disorders show lower HRV, which appears to be a biomarker for psychological disturbance. Detecting lower HRV does not imply a specific diagnosis; rather, it indicates a broad vulnerability to psychological disturbance. Monitoring HRV can serve as a tool in monitoring progress in therapy, with increasing HRV suggesting improved emotion regulation and social connection.

## 9. Heart Rate Variability, Vagal Activity, and Optimal Psychotherapy

Several authors have presented evidence that higher HRV is accompanied by greater social engagement, emotional regulation, sense of safety, and psychological flexibility [18,33,40,41,42,43,44,45,46]. Each of these characteristics is regarded as a desired outcome of successful psychotherapy. As Petrocchi and Ottaviani observed, “It becomes clear that HRV is associated with a number of psychological and behavioral variables that are usually the target of psychotherapeutic interventions” (2024, pp. 375–376) [47]. In turn, psychotherapy proceeds more effectively, when these characteristics are to some degree present.

Elsewhere, I have summarized this research in detail [35]. Here, we will briefly summarize the evidence that higher HRV facilitates the following: (1) social engagement, compassion, and prosocial behavior, (2) autonomic nervous system regulation and emotion regulation, (3) sense of safety, and (4) cortical efficiency and cognitive flexibility.

## 10. Social Engagement, Prosocial Behavior, and Compassion

Stephen Porges (2009, 2023) introduced the polyvagal theory, an account of the emergence of the parent-infant caring bond in the course of mammalian evolution [18,48]. Porges (2009) described the evolving vagal system as a physiological foundation supporting this caring relationship and supporting the compassion and empathy essential to the caring relationship [48]. The caring relationship is reciprocal; the two parties co-regulate with one another emotionally (Porges’ polyvagal model has been challenged, particularly some of his claims about the evolutionary branching of the vagal nerve [49,50]. Porges has responded at length to criticisms and pointed out that many of the criticisms reflect misunderstandings or misrepresentations of the polyvagal theory [51,52]).

Gilbert (2014, 2024a) conceptualized psychotherapy as fundamentally a process of restoring compassion, first in the therapeutic relationship and then in everyday life [40,53]. Activation of the vagal system and increases in HRV support the development of caring and compassion. In turn, the cultivation of compassion moderates any sense of threat and re-activates the vagal system.

## 11. Autonomic Nervous System Regulation and Emotion Regulation

Porges (2009, 2023) describes the calming and soothing effects of vagal activity [18,48]. Parasympathetic influences produce both physiological relaxation and interpersonal comforting. Thayer and Lane (2000) and Thayer et al. (2009) go further, proposing the neurovisceral integration theory, which emphasizes the integration between the autonomic nervous system with the central nervous system [46,54]. The orbitofrontal cortex or more broadly the prefrontal cortex, the executive control center in the brain, regulates both the heart and emotion. When the parasympathetic system is activated, this inhibitory function is optimal, and the subcortical emotion centers are regulated. When the inhibitory functions of the prefrontal cortex are impaired, the emotion centers are dysregulated and negative emotions prevail. Wendt and Thayer (2024, p. 83) concluded that vagally mediated HRV is an “index” of prefrontal cortex functionality [33]. When HRV is high, the autonomic system and the emotion centers are well regulated; when HRV is low, dysregulation prevails (See also Sargent et al., 2024 [45]).

## 12. Sense of Safety

Both Porges and Thayer described a reciprocal two-way relationship between vagal activation and a sense of safety [18,46,55]. On the one hand, both adverse childhood experiences and current life stress can disrupt compassionate engagement. A sense of safety is critical for relational trust and empathy to operate. On the other hand, cultivating compassion and higher HRV serves as a buffer against current stress.

Gilbert (2024b) emphasized the role of “caring circuits” in our physiology, dominated by vagal/parasympathetic activity, in learning a sense of safety and resting largely on interpersonal trust [41]. The therapist who seeks to create a safe place in therapy presupposes a neurophysiology open to safety. Gilbert (2024b) emphasized the value of augmenting verbal reassurance with mind–body strategies such as breath training, imagery, and compassion training to facilitate this sense of safety [41].

## 13. Cortical Functionality and Psychological Flexibility

Ottaviani (2018), Ottaviani et al. (2016), and Wendt and Thayer (2024) documented that lower HRV is consistently associated with rumination, perseverative cognitions, and worries about the future [33,43,56]. These forms of cognitive inflexibility are accompanied by both lower vagal activity and reduced prefrontal connectivity with the amygdala. When HRV and vagal activity are lower, the executive control centers engage less with the limbic centers and cognition is dysregulated. Ottaviani (2018) concluded that this cognitive dysregulation creates risk for both medical and psychological disturbance [43]. Daily worry, in particular, is strongly associated with low back pain, neck pain, respiratory distress, and stomach distress [43].

On the other hand, as discussed in earlier sections, higher HRV and the associated higher vagal activity are accompanied by increased connectivity between the prefrontal cortex and the limbic brain. When optimized, the prefrontal cortex inhibits the fear response, enhances vertical top-down (Top-down neural regulation refers to higher brain centers such as the cortex exercising regulation over lower brain centers. In this case, the prefrontal cortex inhibits limbic brain activity.) regulation of limbic areas of the brain, and cognition is more flexible. Forte et al. (2019) conducted an extensive review of studies on the relationship between HRV and cognition function [57]. They regard the evidence as supporting the neurovisceral integration theory, linking vagally mediated HRV with cognitive functioning. The authors concluded that “The analyzed studies found that a higher HRV, both in time and frequency domains, were associated with finest cognitive performance …” even after adjustments for other variables such as age and years of education [57].

## 14. There Are Many Pathways to Enhance Vagal Activation: Biofeedback Training and Other Interventions to Increase HRV

We have summarized the evidence that subjects with higher HRV show better social engagement and compassion, emotion regulation, sense of safety, and cognitive flexibility. These variables are conducive to effectively engaging in psychotherapy and hypnotherapy. Further, successful psychotherapy and hypnotherapy seek to produce more of these characteristics. This understanding suggests the value of increasing levels of each of these characteristics by self-care practices and lifestyle before and during therapy.

Biomedical research largely treats HRV as a given. Lower HRV predicts disease risk; higher HRV predicts optimal functioning. Lower HRV predicts higher risk of death; higher HRV indicates resilience and longer life. In behavioral health, in contrast, we treat HRV as a variable to be modified by a variety of behavioral and lifestyle interventions [58]. This strategy can optimize psychotherapy and hypnotherapy.

There are many pathways to enhance HRV levels prior to and during treatment. Wendt and Thayer (2024) [33] concluded the following:

The most investigated methods to increase vagally mediated HRV are exercise interventions, HRV biofeedback, and slow breathing interventions, meditation- and mindfulness-based interventions (MBIs), and supplementation of omega-3 fatty acids [33].

In the following, several strategies for increasing HRV will be briefly described. The first, HRV biofeedback, requires additional specialized training. The other strategies are within the scope and training of most hypnosis practitioners. See Table 3 for information on the use of these techniques by hypnosis practitioners.

## 15. HRV Biofeedback

The best-documented behavioral intervention to increase HRV and vagal activation is HRV biofeedback [21,22,59,60]. Padilla et al. (2025) proposed using HRV biofeedback training as an adjunct to hypnosis sessions [61]. The most widely used HRV biofeedback protocol, the Lehrer–Vaschillo protocol, identifies the resonance frequency and guides the individual to breathe at this rate, increasing heart rate variability [19,20,62]. Training begins with a resonance frequency assessment. The practitioner guides the client to breathe for two to three minutes at rates between 4.5 and 7.5 breaths per minute and determines the breathing rate with the largest heart rate variability, the largest oscillations in heart rate.

Once the resonance frequency is determined, the treatment shifts to the patient practicing smooth, paced breathing at the resonance frequency breath rate. The resonance frequency practice exercises can be guided in the office with sophisticated biofeedback systems or guided at home with breath pacers and user-friendly consumer-grade HRV instruments.

In the past, assessing resonance frequency required skilled clinicians and expensive multi-modal biofeedback systems. Biofeedback practitioners have optimally used multi-modal biofeedback systems, with good signal quality, capable of monitoring both respiration and heart rate. FDA approval and ISO 13485 device certification assure quality. Tracking of respiration along with heart rate enables practitioners to analyze HRV changes that are specifically driven by respiration.

Today, automated algorithms working with inexpensive consumer-grade HRV monitoring systems—for example, the optimal HRV™ system (https://www.optimalHRV.com, Accessed on 1 February 2026)—open the possibility for a practitioner to approximate the resonance frequency without advanced training and at low cost. One study used the app-based optimal HRV system with an eating disorders population and found significant therapeutic benefit [63]. Further research is needed to document both therapeutic efficacy and the reliability of automated assessment by comparison to assessment by a trained practitioner.

**Paced slow breathing training.** Ideally, practitioners will guide patients to breathe at their precise individualized resonance frequency for optimal HRV. However, the average resonance frequency for adults is approximately six breaths per minute and several research studies have documented increases in HRV with slow, smooth, gentle paced breathing at about six breaths per minute [64]. Most hypnosis practitioners have experience with guiding patients to a greater awareness of gentle relaxed breathing as part of the hypnosis process [65]. Adding a deliberate pacing at six breaths a minute, using a smart phone breath pacer or a consumer-grade device such as the Inner Balance™ (https://store.heartmath.com/innerbalance, Accessed on 1 February 2026), will serve to better assure an increase in HRV. Practicing six breaths per minute breathing prior to commencing hypnotherapy and during psychotherapy promises to enhance the therapy process. This author integrates a brief period of gentle paced breathing with the patient at the beginning of most hypnotherapy and psychotherapy sessions.

**Music-based interventions.** Our nervous system entrains to a variety of inputs that carry rhythms relevant for brain activity or cardiovascular activity. There is an extensive published literature on sound and light stimulation with beneficial effects on neural regulation [66,67,68]. A number of studies have also explored the effects of music-based stimulation oxytocin release. Chu and Tsai (2026) reviewed 20 studies and found peripheral oxytocin increased with many short-term music interventions, supporting social bonding and emotion regulation [69]. These findings were context-dependent and not found with all populations.

**Diet.** Nutritional choices influence HRV in a variety of ways. Most immediately, obesity is strongly associated with lower HRV. Young and Benton (2018) conducted a review of relevant research and concluded that in general weight loss increased HRV [70]. They also found evidence that the nature of the diet affects HRV. Their study suggested that following the Mediterranean diet increased HRV. A variety of specific dietary elements also tended to increase HRV, although recommending specific dietary elements remains the provenance of a dietician. Nutritional choices should be influenced by a number of factors and should be individualized, since not all individuals show benefit from the same regimens [71].

**Exercise.** Exercise has long been regarded as a core factor in healthy lifestyle. Sedentary lifestyle is associated with lower HRV values as well as obesity [72]. Intense athletic training produces increases in sympathetic activation, increases in HR, and reduced HRV. One early study showed a more rapid recovery of HR and HRV after exercise trials in athletes compared to sedentary controls, indicative of higher resilience [73].

More moderate exercise programs, such as those in cardiac rehabilitation, produce increases in HRV and vagal activation [74]. Recent studies have documented that exercise can increase baseline HRV in individuals with serious chronic conditions including diabetes and cardiovascular disease (See an extensive review by Routledge et al., 2010 [75]). Routledge et al. (2010) hypothesized that exercise that increases HRV serves to improve vagal modulation and reduce sympathetic activation [75]. Encouraging increased exercise activity in potential therapy patients will have a variety of beneficial health effects and serve as well to increase HRV and enhance the therapeutic process.

**Meditation and mindfulness training.** A number of researchers have reported increases in HRV during and/or after meditation, utilizing mindfulness meditation, Zen meditation, breath meditation, and other techniques [76,77]. One recent systematic review reported evidence that brief mindfulness meditation increased RMSSD, an HRV statistic and marker for parasympathetic activation [78]. Teaching patients simple mindfulness and meditation techniques as adjuncts to their therapy and encouraging regular meditative practice will serve to enhance HRV with potential benefits for therapy and for emotional health in the long term.

**Self-hypnosis practices.** Diamond et al. (2008) reported a strong correlation between hypnotic depth and HRV [79]. The more deeply the research subject went in trance, the higher the amplitude of high-frequency HRV exhibited. Chen et al. (2017) measured HRV before, during, and after hypnosis interventions for depression, and they found increases in several HRV indices, including RMSSD during and post-hypnosis [80]. This serves as a supplement to the in-session hypnosis interventions, augmenting the autonomic effects. Training patients early in treatment in self-hypnosis and encouraging regular practice during treatment may enhance psychotherapy or hypnotherapy, providing the patient with a lifelong mind–body coping skill.

## 16. Limitations

There is an abundance of published studies, as cited above, documenting the correlations between low HRV and medical conditions, especially chronic illnesses such as diabetes, heart disease, hypertension, and cancer. There is also a large and growing body of studies showing the correlation between low HRV and psychological conditions, especially anxiety disorders, major depression, and post-traumatic stress disorder. This research is sufficient that Beauchaine and Thayer (2015) called HRV a “transdiagnostic marker for psychopathology.” [34].

The body of research assessing whether increasing HRV moderates disorders is weakened by methodological weaknesses and small-sample studies. Nevertheless, a careful meta-analysis by Lehrer et al. (2020) concluded that the published studies on both HRV biofeedback and six breath per minute paced breathing show modest effects sizes (Hedge’s g) comparable to those for other effective behavioral health treatments [22]. The effects sizes were largest for anxiety, depression, anger, and artistic performance, and smaller but still significant for PTSD, sleep, and quality of life. Nevertheless, more research with larger samples is necessary to assess the therapeutic effects of HRV training and paced breathing.

The evidence that higher HRV correlates with social engagement, emotion regulation, sense of safety, and psychological flexibility is moderately strong, leading to the conclusion by Petrocchi and Ottaviani (2024) that higher HRV is associated with a number of psychological and behavioral variables that are the target of psychotherapeutic interventions [47].

The greatest limitation for the present review lies in the lack of empirical studies assessing the link between interventions increasing HRV and psychotherapeutic/hypnotherapeutic outcome. Caldwell and Steffen (2018) documented that adding HRV training to psychotherapy produced a greater reduction in depressive symptoms than psychotherapy alone [81]. No similar studies have yet assessed the outcome of adding HRV training to hypnotherapy. More research is needed on the benefits of adding HRV training to both psychotherapy and to hypnotherapy.

The adverse effects of biofeedback and breath training are minimal, but caution should be exercised when applying these therapies in individuals with labile medical and psychological disorders. Medical consultation prior to and during treatment is recommended. For example, patients with diabetes may require medication adjustments if treatment is successful; stress reduction sometimes moderates blood sugar levels, requiring less insulin (McGrady & Lakia, 2016) [82]. Similarly, biofeedback and breath training may trigger dissociative reactions in patients with dissociative and post-traumatic psychological disorders.

## 17. Conclusions

Hauk and Kritikos (2018), Lehrer (2024, 2025), Steffen and Moss (2024), van der Kolk (2015), and many others have argued that psychotherapy will benefit from greater attention to embodiment and psychophysiology recently demonstrated that adding HRV training to psychotherapy increased the effectiveness of depression treatment [81,83,84,85,86].

Heart rate variability (HRV) is a critical dimension in physiology, closely connected with autonomic balance, limbic modulation, emotion regulation, and social engagement. The present article introduces a basic understanding of heart rate variability, summarizes its role in medical illness and psychological disturbance, and proposes that strategies that increase HRV offer one means to optimize the process of psychotherapy and hypnotherapy.

Mind–body therapies, like all behavioral health therapies, require a thorough initial evaluation to identify medical problems, such as untreated cardiopulmonary illness, diabetes, or mental illnesses, that require a medical referral and ongoing management. Once the patient is cleared for behavioral health care, attention to HRV can enhance the treatment process.

Therapists can harness the brain and nervous system to advance therapeutic success by directly training HRV via biofeedback, introducing prospective clients to simple self-care strategies such as slow breathing and self-hypnosis, and encouraging health promoting lifestyle modifications that support higher HRV. Introducing these HRV-enhancing activities at the outset of treatment may enhance the treatment process. Utilizing these activities intermittently during the course of therapy will further optimize the process.

For therapists who lack the skills to introduce meditation, mindfulness, and other self-care practices, referral to local specialists or classes in each area will accomplish the same objective. There are also an abundance of training apps available online, as summarized in recent reviews [87,88,89]. These positive steps will aid psychotherapy and also provide long-term health benefits for the years after therapy.

## Figures and Tables

**Table 1:** Commonly used time-domain statistics for heart rate variability.

SDNN—the standard deviation of the normalized time interval between heart beats.
RMSSD—the root mean square of the successive differences in time intervals between heart beats.
PNN50—the percentage of successive interbeat intervals that differ from adjacent intervals by 50 milliseconds or more.
HR Max minus HR Min—the average difference between the highest and lowest heart rate within each respiratory cycle.

**Table 2:** Frequency ranges of HRV.

The standard definitions of the frequency ranges for HRV were established by the Task Force (1996) [8]:
Ultra-low frequency range—<0.003 Hz
Very-low frequency range—0.003–0.04 Hz
Low frequency range—0.04–0.15 Hz
High frequency range—0.15–0.4 Hz

**Table 3:** Clinical hypnosis practice and interventions to enhance heart rate variability.

HRV Biofeedback—Requires a trained biofeedback practitioner and a multi-modal biofeedback system. Hypnotherapists can seek biofeedback training or refer the patient to a trained biofeedback practitioner. Once a resonance frequency has been determined by a biofeedback therapist, the hypnotherapist can utilize a brief period of resonance breathing at the outset of each session.
Paced slow breathing—Requires experience in diaphragmatic breathing training and the use of a breath pacer or breathing app to guide breathing at six breaths per minute.
Music-based interventions—Exact determination of optimal musical prescriptions for an individual remain the provenance of a music therapist. Hypnotic practitioners can recommend attention to soothing, grounding music and provide soothing music in the clinic.
Diet—Development of a specific plan for dietary modifications is the task of a nutritionist or dietician. Hypnosis practitioners can refer patients for nutritional evaluation, encourage patients to follow a generally healthy diet, and facilitate the patient’s compliance with a currently prescribed dietary plan.
Exercise—Hypnosis practitioners can encourage the patient to set small stepwise goals to gradually increase activity. Practitioners can also refer patients to group-based exercise programs supervised by exercise physiologists, physical therapists, or other trained professionals.
Meditation and mindfulness training—Hypnosis practitioners will benefit from learning a handful of meditation techniques along with mindfulness strategies. Meditating briefly with a patient to open the therapeutic session will enhance openness for therapy and provide the patient with a lifelong coping skill.
Self-hypnosis—Most hypnosis practitioners learn a variety of self-hypnosis techniques during their hypnosis training. Teaching patients one or more self-hypnosis techniques and encouraging regular practice will contribute to increased HRV.

## Data Availability

No new data were created or analyzed in this study. Data sharing is not applicable to this article.
